# Extracts and Gleanings

**Published:** 1875-10

**Authors:** 


### Extracts and Gleanings.

---

*Messrs. Editors:* Through your valuable journal, permit me to direct the attention of the profession to the positive virtues of the cyprepeduem, or ladies' slipper, as an emmenagogue. We all learned to doubt the existence of any remedy which claimed to be especially emmenagogue; and how could we help it, when such men as Meigs taught that there was no such thing as a direct emmenagogue?

My attention was accidentally called to the ladies' slipper in 1850. Having at that time an epileptic female patient, and in searching for something new, my eyes fell upon the cyprepeduem, as described by the United States Dispensatory. It was recommended as having occasionally been beneficial in epilepsy. It was procured and prescribed for my patient, and although there seemed no change whatever in the epileptic attacks, yet the patient was relieved from an obstinate amenorrhœa of several months' standing.

This case induced me to try thoroughly the remedy as an emmenagogue. There is a very decided difference between the plant which I call ladies' slipper, and the description of the same plant by Wood and Bache.

Mr. Editor, have you never seen the flower which I call ladies' slipper? Indeed, it has not much the appearance of a slipper, but bears a striking resemblance to the vulva in form, in color, and general arrangement; and if the labia are gently separated, you may not draw very greatly upon your imagination to see clearly a very fair representation of penis and testicles.

My experience with the root is most satisfactory, and when we consider the great and most striking resemblance between the flower and the genito-urinary organs, may we not conclude with some propriety, that nature speaks and points to the flower as a remedy in all diseases of those parts. It is with no little confidence that I recommend its use to the profession in all cases of amenorrhœa and dysmenorrhœa as a remedy in secondary syphilis, chronic rheumatism and cutaneous effections it will gratify any practitioner. The preparation is innocent, and may be used as a cosmetic, in doses of ʒj. three times a day, the skin clears up, and a beautiful complexion is the result most generally.

My first preparation was from root gathered myself, and made into a tincture. It can be procured from Dr. G. C. Starke, a druggist of Petersburg, Va., who has made a fluid extract from root collected myself.

Dr. Starke informs me that many prominent physicians are prescribing it with satisfaction, and I earnestly request that the profession may give it a trial, and report results in your journal.

In a few days you will receive a bottle, and it is to be hoped that you will give the extract a trial in your own practice.

Dose—ʒj. three or four times a day, beginning one week before menstruation is expccted, and continuing the dose during that period once every four hours. For secondary syphilis, cutaneous affections, etc., ʒj. three times a day. I must close, and shall look confidently, expecting to hear from you, and also from your correspondents. Very truly your friend,

G. H. Batte, M. D.

*Rural Bower, Greensville Co., Va.*

We have known Dr. Batte from boyhood. He is a man of an extraordinary mind, and we can endorse for the truth of all he says. We have never seen the Doctor's ladies' slipper. With his description we think, however, we would recognize it, and we agree with him in the opinion that "nature speaks and points to the flower as a remedy in all diseases of those parts." We would advise our readers to get their home druggist to order a few bottles from Dr. G. C. Starke, a druggist of Petersburg, Virginia. Dr. Starke was also a *school-mate* of ours, and is an educated gentleman. We hope Dr. Batte will favor us with another article. P.

*The Necessity of Active Treatment in all Acute Diseases of the Throat.*—The variety of throat affections of a grave type, which frequently make their appearance when there is no epidemic of that kind prevailing, should admonish all practitioners the necessity of a prompt and energetic treatment of all cases of acute diseases of the throat.

Admitting, then, that sore throat, even of a mild character, should not be neglected, the question of treatment requires consideration. There is strong reason to believe that scarlatina and diphtheria are connected essentially or indirectly with the development of germs, and the local remedies used should, therefore, be taken from the class of antiseptics. Such remedies were used long before the germ-theory was accepted with any favor, apparently because they were found to be of use. Whilst I shall not attempt to enumerate the number of remedies of the class referred to, I wish to call the attention of the profession to the claims of salicylic acid, an article highly extolled in a recent number of the MEDICAL RECORD, and if entitled to half the praise bestowed upon it, as an anticeptic, is certainly worth a trial.

The main point, however, which it is proposed to present in this article is, that no acute sore throat should be neglected, however mild, and that antiseptics should form a part of the loeal treatment. Such attacks should not be neglected, both because they may be essentially grave diseases, and also because, if treated very early, early resolution may and often follow. A. G. SMYTHE, M. D.

*Baldwyn, Miss., April* 30, 1875.

It is due to Dr. Smythe to say that the above article should have been published in the May number before a similar suggestion appeared in the RECORD. P.

*Wet Strapping.*—It has been regarded as an item of some importance in the treaement of old ulcers, such as are of specific nature, found upon the lower extremities, that, if strapped, the plaster should be permitted to remain as long as possible without change. Whether the strapping meets the indication or not, cleanliness, etc., are, of course, to govern the surgeon in every case. With this object in view, several cases have been dressed with what have been called wet straps, which are prepared by passing strips of adhesive plaster through hot water instead of heating them in the usual manner. In these instances the water was also carbolized.

It seemed quite certain that the ulcers had healed more rapidly under this plan of treatment than any which had been adopted, and it was very evident that the plaster did not get loose as quick as when heated over a spirit lamp.—*Medical Record.*

*Ovariotomy Complicated with Pregnancy—Cæsarean Operation—Cure.*—Mr. Thomas Hillas, at a meeting of the Medical Society of Victoria, Australia, held December, 1874, (*Australian Medical Journal*, February, 1875,) gave this account of the following case: The patient, aged twenty-four years, single, was admitted to the Ballarat District Hospital, June 4, 1872. The history of her case was peculiar. She believed that she became pregnant in March, 1871. She was admitted to the lying-in ward of the Ballarat Benevolent Asylum in November, 1871, and after staying there until the following June, a consultation was held, and she was discharged, her case being deemed ovarian dropsy, and not pregnancy. On her admission to the hospital, she was examined by the staff, all agreeing that she was suffering from ovarian dropsy, and that it was a suitable case for operation. On June 13th, the patient being chloroformed, Mr. Hillas commenced the operation by an incision midway between the umbilicus and pubes. Mr. Hillas says: "On arriving at the peritoneum, I made a small opening into it, when out spurted a large jet of venous blood, which the pressure of the finger controlled. I came to the conclusion I had wounded, unwittingly, a gravid uterus, and feeling sure of this, I extended the first incision upwards to the umbilicus, when a large uterus rolled out on to the thighs, and the ovarian sac protruded." The latter was tapped, and about eleven quarts of fluid withdrawn. The pedicle was short and thick, and after being tied firmly with a double whip-cord ligature, the clamp was securely applied, and the pedicle divided, the ends of the double ligature being tied over the ends of the clamp. The question then arose, what was to be done with the uterus, which was all this time lying on the thighs, with a fœtus in it, and a wound through its muscles, probably into the placenta.

Some advised that the wound should be sewn up, and the organ replaced in the abdomen; but seeing that labor must soon come on, and thinking that rupture of the uterus would most likely occur at the seat of injury, Mr. Hillas decided to perform the Cæsarean operation, as being the most likely means of giving the patient a chance to recover. The uterus was incised to about five inches, and the placenta and a fœtus, alive and well developed, at about the eighth month of gestation, extracted. The wound in the uterus was then closed with silver-wire sutures, the cut ends being carefully tucked down into the incision. The uterus then contracted firmly. The wound of the abdomen was then closed, the clamp being left at the lower margin of the wound, and a good deal dragged upon. The right ovary was the one affected. The patient measured sixty inches around the abdomen before the operation. The sac and its contents weighed thirteen pounds. The patient vomited for about forty-eight hours after the operation, having been an hour under chloroform. This was relieved by morphia and ice, and on the fourth day all unfavorable symptoms abated. There was a discharge of pus from the lower portion of the wound, which ceased in about a fortnight, and then it completely healed. She was discharged, cured, at the end of six weeks. On July 3d, a month after the operation, she menstruated moderately for four days, and again on August 28th.

Mr. Hillas has seen her several times since, and reports her to be in perfect health.—*Medical Record, July* 31, 1875.

*Perineal Tumor of Fœtus an Impediment to Delivery.*—Mrs. A. B., aged twenty-two, of good constitution and excellent health, who was delivered of her first child a year and a half ago with forceps, by Dr. C. D. Homans, after a hard labor, expected to be confined about the first of February, 1875. She had not menstruated since the birth of her first child, a well developed and healthy infant. On January 28th, I was called to see her, and found her with a pulse of 130; the temperature was 103°; the respiration was rapid; cough, rachitic pains, considerable prostration, pain of throat and neck, and slight redness of the fauces. The next day there was a pultaceous patch the size of a little finger-nail on the left side of the uvula. This disappeared in twelve hours under the use of a lotion of chlorate of potassa. Wine and quinine, with frequent and nourishing food, were prescribed, and on February 1st she was nearly well.

During pregnancy she declared that the child was not carried like the first: she had had no fright, nor had she seen anything to annoy her. There were no twins nor monsters known in her family or in that of her husband. About February 18th she ceased to feel the motion of the child; a cold weight took its place, and she affirmed it was not living.

At 8 P. M., of February 24th, labor began; the face presented, left mento-anterior; the cranial bones crepitated on pressure. The membranes having been ruptured, the pains became efficient; a little ether was given as they increased; the head was born easily, the shoulders with much effort. When the thorax had been delivered, no further progress was made; the abdomen felt as though another child was present. On introducing a hand along the child's back, an obstruction was discovered above the brim, where the breech of the fœtus was separated by a sulcus from another body swelling beyond it. Dr. Miller now saw the patient. The perforator, being carried up between the fœtal body and the operator's hand, was thrust into the tumor and gave vent to a prodigious burst of blood and water. Vigorous traction now brought away the child. The uterus, followed down by the hand, contracted firmly, and no hemorrhage of moment ensued; the placenta came away directly. It was now seen that labor had been impeded by the growth upon the child's breech of a half-solid tumor, larger than the fœtal head. The incisions made by the perforator having been sewed up, the tumor resumed its size after stuffing, and a cast was taken by Dr. Bolles, who has placed it in the pathological cabinet of the City Hospital. He describes it as follows: "The tumor was situated behind the anus, and distended it somewhat. It evidently arose behind the rectum in the lower part of the pelvis; it was not attached to any of the pelvic organs, but could be easily and cleanly dissected away from them all. It was chiefly a large cyst which had been ruptured in delivery, and apparently contained degenerated blood. Into this cavity there projected from above several smaller tumors, in all a mass about three and one-half inches in diameter, the largest of which contained grumous fluid. The others were semi-solid, dark and pulpy." There was no evidence of fœtal inclusion. The tumor appeared to be glandular. Its largest circumference (antero-posterior) was sixteen and one-half inches. The gland of Luschka is referred to as the origin of such growths.—*Boston Medical and Surgical Journal.*

*Failure of Bromide of Iron in Chorea.*—Chorea, like the correlated disorder, rheumatism, has suffered many things of many remedies. It is certainly a disease which can be favorably influenced by different drugs and by various modes of treatment. Bromide of iron has lately been strongly recommended for it by Professor J. M. Da Costa. In a lecture on chorea, reported by Dr. F. Woodbury, in the *Medical and Surgical Reporter* for January 30, 1875, referring to bromide of iron, Prof. Da Costa says: "Having now used it for three or four years, my experience from the treatment of a large number of cases giving abundant opportunity to witness its good effects, induces me to like it better than any other one article in the treatment of chorea."

Since reading Professor Da Costa's remarks, I have tested the remedy in twelve cases of chorea, chiefly in the clinical service of Professor H. C. Wood, at the Hospital of the University of Pennsylvania, and have been entirely disappointed in the preparation. In no case was any permanent benefit derived from its use. In two instances slight apparent improvement was noted, but it was only transient in character. Much better results can be obtained from other salts of iron, and from other remedies, such as arsenic, cimicifuga, and the bromide of potassium.

It was usually given in plain syrup and water, commencing with five grains three times daily, as recommended, and rapidly increasing the dose to twenty. The treatment was continued from two to four weeks. Twenty grains very generally caused vomiting. It seems to be a remedy which quickly irritates the intestinal tract. In the case employed by Professor Da Costa to illustrate his lecture, this fact was noticed. The child under treatment became affected with a troublesome diarrhœa, requiring the substitution of bismuth for the iron, and several attempts to return to the bromide were foiled by this irritability of the digestive tract, which refused to tolerate the drug. Fowler's solution was finally given in its place.

Most of the cases in which the bromide of iron failed were afterwards cured either by arsenic, arsenic and iron, or cimicifuga, assisted by hygienic measures. In local chorea or clonic muscular spasm, for which this remedy is advised after the failure of other methods of treatment, I have not found it of any value. Very rarely indeed will any drug improve these local affections.

Dr. J. B. Rudderow informs me that he has used the preparation under consideration without success in several cases of chorea.

In addition to the twelve cases of chorea referred to, I have also unsuccessfully employed the bromide in a few cases of hysteria and epilepsy. Theoretically, the combination of bromide and iron would appear to be a good one for the treatment of a neurosis like chorea, and it is well known that any of the numerous remedies which have been employed successfully in this disease may sometimes prove futile; but the failure of the bromide of iron has been so marked in my experience that I have thought it worth while briefly to call attention to the fact.—CHARLES K. MILLS, M. D., in *Medical Times.*

*Carcinoma* (*New York Medical Journal, September,* 1875; *from Med.-Chir. Centralblatt,* 20, 1875.)—Professor Von Nussbaum, after treating over one thousand cases of cancerous disease, has arrived at the following conclusions:

1. Cancer is a proliferation of the epithelium, which progresses rapidly, and crowds out the connective-tissue stroma, undergoes ulceration from slight causes, determines local destruction, induces severe general illness from hemorrhages and discharges, and finally disseminates its particles throughout the whole body, and generates an identical proliferation and destruction in various organs, and thus destroys life.

2. Its causes are found in advanced age, grief and care, and when there is a disproportion between epithelium and connective-tissue—warts, cicatrices, glandular swellings, etc.; and, lastly, those tissues are more especially affected which are subjected to frequent irritation, though not placed in a condition of acute inflammation. Cancer is not congenital or contagious. At first it is a purely local disease, which only becomes a dyscrasia by the dissemination of its elements throughout the body.

3. The humoral infection must be distinguished from the dyscrasia. The former can disappear entirely, and never contra-indicates the operation.

4. Recurrence of cancer is either continuous, when cancerous elements have remained, or regionary, when neighboring tissue disposed to cancerous disease has remained; or it may be a recurrence by transplantation, when cancerous particles have been disseminated by passing into the circulation through opened vessels.

5. Cancer can be cured radically by early and extensive operation.

6. Exact and extensive statistics show that patients who are operated upon live longer than those who refuse all interference.

7. In the treatment, all remedies which act on the tissues, blood and nerves, come into consideration. Early and extensive operation ranks first. Caustics are often useful, especially after the cancer has been scooped out. Parenchymatous injections deserve trial.—*Medical Times.*

*Removing the Ovum in Cases of Abortion.*—Dr. Paul F. Munde, in a letter to the *New York Medical Record*, calls attention to Hoenig's method of removing the ovum in cases of abortion. Hoenig recommends to express the ovum, either entire or in part, if the fœtus be already removed, by means of bi-manual compression, two fingers of one hand being introduced into the vagina, and passed as far up as possible into the fornix vaginæ, the other hand grasping the uterus through the abdominal parietes, thus firmly compressing the organ between the fingers of both hands, and slowly and surely expelling its contents. If the uterus is anteverted or anteflexed, as usual during the earlier months of pregnancy, the two fingers should be passed into the anterior cul-de-sac, or the corpus uteri may be firmly pressed against the symphysis pubis by the external hand alone (the bladder having been emptied). If the uterus is retroflexed, the two internal fingers go behind the cervix. Of course, it is essential that the cervix be sufficiently dilated before this method is attempted.—*Boston Medical and Surgical Journal.*

In the January number, 1874, of this journal, we gave our experience in regard to the practicability of removing the ovum, or the after-birth, in cases of abortion, by a bi-manual operation. It differs from the above method of Hoenig's only in the fact that while he claims that the contents of the uterus may be moved or forced out by bi-manual compression, we the more certainly remove them by the introduction into the uterus of one or more fingers of the right hand, while the uterus is forced down and held firmly, by pressure above the pubis, with the left hand, the patient being first propped up in bed to an angle of 45°, and the heels drawn up to the buttox to relax the abdominal walls. This method we have successively practiced since the year 1856. While superior excellence is claimed by Dr. Hoenig for his plan, cases will often occur wherein the secundines, being too firmly attached, cannot be thus pressed out, but must require to be detached and *scooped out* with the finger, as by the method we suggest. R. C. W.

*Oxide of Zinc in Infantile Diarrhœa.*—Dr. Fogel (Academy of Medicine, Cincinnati,) reported excellent results from the administration of oxide of zinc in one-half to one grain doses in the diarrhœa accompanying teething, and in the chronic indigestion of infants. He had encountered several cases of these affections occurring in children, especially those artificially fed, in which this remdy effected a cure after all other means had failed. He generally combined it with pepsin and bismuth, but did not consider the good effects due to the latter agents, as he had tested its efficacy on several occasions by omitting it, when the symptoms recurred with more or less severity.

Dr. Whittaker had had some experience with the oxide of zinc in infantile diarrhœa. He usually gave it in grain doses, and was pleased with its action, but usually preferred bismuth in the form of the subcarbonate and subnitrate, though it s›metimes causes severe vomiting from adulteration with arsenic.

Dr. Conner said that seven years ago he had examined eight or ten specimens of bismuth obtained at different drug-stores of the city, nearly one-half of which contained an appreciable amount of arsenic. The injurious effects may sometimes be dependent upon this impurity.

Dr. Bartholow stated that if these preparations are made according to the formula laid down by the United States pharmacopœia, they will be freed from any arsenic the bismuth may contain.

Dr. Reamy had used the oxide of zinc with good effect, but preferred bismuth and pepsin. He usually gives 3-7 grains of pepsin and one-half that amount of bismuth every four hours to children one to two years old. He had found these agents of signal service. Most cases of that fearful malady, enterocolitis, are primarily simple diarrhœa, which in the first and second stages will generally be promptly controlled by pepsin and bismuth. The local action of bismuth is very mild, no injection in gonorrhœa being better than one containing bismuth. He would attribute good results in Dr Fogel's cases, chiefly to the pepsin prescribed with the oxide of zinc• *The Clinic.*

THERAPEUTIC NOTES.—*Anti-Catarrhal Pills.*—

℞.—Gum. ammonic. . . . . . gr. xxx.
Ammonii carbonat. . . . . gr. xv.
Ipecac. pulv. . . . . . . gr. iii.
Morphiæ muriat. . . . . gr. iss.
Mucilaginis. . . . . . q. s. M.

Ft. in pil. no. x. Coat with balsam of tolu dissolved in chloroform. Take one pill morning and evening in chronic bronchitis.

*Dressing for Ulcers.*—

℞.—Olei cadini. . . . . . ʒss ad. ʒi.

Pulv. calci. sulphat. . . . ℥vj. M.

To be thinly spread on dressing for ulcers, and renewed three, four, five or six times daily, taking care to cleanse the ulcers after the dressing has been removed. Of especial value in the treatment of profuse and fetid suppuration.

*Treatment of Lead-Colic by Chloroform.*—Chloroform has lately been used in lead-colic, both externally and internally (by inhalation,) with advantage. Dr. Barduzzi has employed the following formula with success in one case which had resisted other forms of treatment :

℞.—Chloroformi. . . . . . ʒi.

Syr. acaciæ. . . . . . ℥ii ʒvii.

Aq. destillat. . . . . . ad f℥vj. M.

This potion is administered in two parts, with a quarter of an hour interval.

In Dr. B.'s case this potion was given three times in thirty-six hours, with the effect of relieving the patient greatly.

Inunctions with a chloroform embrocation were at the same time employed on the abdomen.

*In Syphilis.*—

℞.—Hydrarg. bichlor. . . . . . . gr. i.
Opii. . . . . . . . gr. ii.
Pulv. glycyrrhizæ,
Ext. gentian., . . . . . . . aa ʒss. M.

Ft. in pil. no. 16. One to be taken morning and evening after eating.—*Medical Times.*

*Tetanus, Twenty-eight Days After Injury, Treated by Chloral.*—On March 4th, J. B., aged 13, whilst slicing roots, cut his forefinger through. As the bone was fairly cut, and the finger was still hanging on by a piece of skin, I determined to save it. I set the bone, and everything went on well until April 2d, when he called on me, complaining of pains all over, and a general feeling of chilliness. As I found he had been sitting about on the ground the greater part of the previous day, I treated him for a severe cold. On calling the next day, I found him in bed, very excitable, and only able to rest whilst lying on his face. The next day, April 4th, no mistake as regards the diagnosis could be made. He remained in much the same state for a fortnight, and then very severe hysterical fits occurred about every twelve hours. These fits lasted about a week, and, after they left him, he made gradual recovery. During the first three weeks he took five grains of chloral in camphor-water every four hours; and, if this mixture were in any way altered, he became more excitable. After that period, I gave him a mixture containing Indian hemp and bromide of potassium. I found all kinds of external applications, as well as hypodermic injection of belladonna, useless; and injections for the relief of the obstinate constipation produced so much excitement that I was obliged to depend on croton oil. Beef-tea, sherry, whipped eggs and milk were taken freely, and the darker and more quiet the room the better. The mother stated that the boy was always very excitable, and every spring suffered from frequent attacks of epistaxis; this year he had an attack nearly every morning through March, but not a single attack during the time he was under my care. DR. STEPHENS in *British Medical Journal.*

*Poisoned by a Hat.*—A peculiar case of poisoning occurred lately at Stettin. On the day before Whit Sunday a shoemaker bought a felt hat. After wearing it, although it did not press on his head, he had severe headache; and an eruption appeared attended with swelling on his forehead, proceeding to suppuration at some parts. The eyes also became inflamed and almost closed; and the swelling extended more or less over the whole face. The hat was placed in the hands of an official analyst, who found that the brown-leather lining was colored with an aniline dye containing poison.—*British Medical Journal, June* 19, 1875.

*Treatment of Diphtheria and Scarlet Fever.*—Dr. G. Mayer (*Fahrb. f. Kinderk.*) directs, even in children under one year, small pieces of ice to be put frequently into the mouth, followed, if possible, every minute or two by a teaspoonful of iced water. In severe cases the external use of cold, by means of an ice-bag applied around the throat, is very useful. The author has found that by this mode of treatment the fever soon diminishes, and the diphtheritic membrane is detached and expectorated. It is only in exceptional cases that the disease extends nevertheless to the larynx. But in one case the author was obliged, in order to reduce the temperature, to resort to cool baths. The latter he also found very useful in scarlet fever. Whenever the temperature exceeds 102° in scarlet fever, the patient is to be placed for ten minutes in a bath of a temperature varying from 93° to 73°, according to the intensity of the fever. The effect of these baths in reducing the temperature lasts for two or three hours.—*Canada Medical Record.*

*Hyperidrosis Cured by the Local Application of Diachylon Plaster.*—Dr. J. M. Bigelow (*New York Medical Journal*, June, 1875) reports a very obstinate case of sweating of the feet that had baffled all ordinary treatment, in which a speedy cure was effected by the local application of diachylon plaster. Putting the patient in bed, he applied the plaster as follows: After cutting it in strips of convenient width he twisted them about each toe separately, and also applied them to the interdigital spaces, completely enveloping the whole foot, so that every portion of the sole, dorsum and toes of the feet was in close and immediate contact with the plaster. These strips were removed each morning, the feet carefully and thoroughly wiped with dry, heated flannel, and new plaster strips applied. For thirteen days this treatment was continued, when the plasters were removed, and the feet presented a healthy, normal appearance, free from the troublesome hyperidrosis. For more than a month the cure has been perfect.

*Incontinence of Urine.*—If they blister the child's sacrum, put it on tinct. of iron and belladonna, and gave no salt in its food, they would have very few cases of incontinence of urine.—T. M. Madden, *Dublin Journal Medical Science.*

*Treatment of Abscess of Breast by Compressed Sponge.*—A patient had been suffering from mammary abscess for three weeks, but without any special benefit from treatment in checking the discharge of pus. It was decided to try the effect of compressed sponge, and for this purpose a sponge about ten inches in diameter was subjected to pressure and then applied by means of a bandage over the breast. After it had been in use forty-eight hours, the abscess was completely cured. No pain was experienced by the patient, and in this case the opening in the breast was three inches above the dependent part of the abscess. In applying a sponge to the breast in this class of cases, it is found of advantage to compress it when dry. After it is applied to the breast and firmly secured in position, a little water is poured upon it to cause expansion and the necessary pressure. *Medical Examiner.*

*Premature Baldness — Treatment.*—Dr. Pincus (*Berlin Klin. Wochenschrift, London Medical Record*) suggests the following treatment in the first stage of premature baldness. This stage is recognized by a daily loss of under fifty hairs, by diminished sensibility to pressure, and, after a time, by commencing hardness and immobility of the scalp. If now weak alkaline washes be applied to the hair for a year or more, the progress of baldness is arrested; and in some cases the mischief already done is restored. He recommends a solution of caustic potash. one part to five hundred of water, or fifteen grains of the bicarbonate of potash to an ounce of water. Two or three drachms of this solution is to be rubbed into the scalp for from three to five minutes daily. After a time this may be done every other day and then only once a week.—*Detroit Medical Review and Pharmacy.*

*Horse-Hair for Sutures.*—Dr. W. W. Green (*Boston Medical Journal,* August 5, 1875) strongly recommends the use of horse-hair for sutures in all cases except where greater strength is required. It is so fine as to leave no scar and to allow the sutures to be introduced very near together, perfectly non-irritating, more easily tied than any other material, and not inclined to slip while tying the second knot. He has never obtained such beautiful, delicate linear scars with any other article. The hair is prepared as follows: It is selected from the tail of a young healthy horse, and first washed in warm water. It is then boiled for half an hour in a solution of soda, strength of an ounce of bicarbonate of soda to two quarts of water. Then it is rinsed in warm water and is ready for use. It does not snarl or kink.—*Detroit Medical Review and Pharmacy.*

*The Value of Tar in Bronchial Catarrh and Winter Cough.*—Drs. Sidney Ringer and William Murrill contribute a note on the use of tar to the *British Medical Journal.* They have employed tar in two-grain doses, made into a pill, every three or four hours. From October to January, inclusive, its effects were watched on twenty-five patients, whose ages varied from 34 to 70. All these patients had suffered several years from winter cough, lasting the whole winter.

These patients suffered from paroxysmal and violent cough, each attack lasting from two to ten minutes—recurring ten or twelve times a day, and breaking their rest at night. Expectoration abundant, frothy, and purulent. Breathing short on exertion, but most could lie down at night without propping. The physical signs showed a variable amount of emphysema, with sonorous and sibilant rhoncus, and occasionally a little bubbling rhoncus at the base. These patients usually began to improve from the fourth to the seventh day; the improvement rapidly increased, and in about three weeks they were well enough to be discharged. The improvement was so decided that even those patients who, in previous years, had been confined to the house during the whole winter, returned to their work. On discontinuing the tar, relapses often occurred in a week or two, but on re-administering the medicine, relief was again obtained.

*Glycerin in Medical Pastes.*—Ferdinand Vigier recommends to add to marshmallow paste and similar preparations some glycerin, to prevent them from getting dry and hard; 1 part of glycerin to 40 parts of gum will be found sufficient for this purpose. This idea had suggested itself to him from the good results obtained by the addition of a little glycerin to pillmasses.—*American Journal of Pharmacy.*

*Incontinence of Urine—Cure.*—A boy, under the observation of Dr. J. J. Reid, had been suffering for sometime with incontinence of urine, and at first it was proposed to try the method of sealing up the prepuce by means of collodion. After examining the patient carefully, it was found that there was dullness over the bladder, extending up for four inches. The boy was then directed to pass his water, and, after passing four or five ounces, a noticeable dullness still existed. The catheter was then introduced, and, by pressure made over the pubes, several ounces more were obtained. The following day he did not suffer from incontinence, and the obvious explanation of the case is, that, from atony of the wall, the bladder could not empty itself, even when assisted by the efforts of the little patient. No stone could be detected, and the urine was in every respect normal. Tincture of nux-vomica was given to him in one-drop doses three times a day. At the present time he is doing well. *New York Medical Journal.*

*How to Cure Pyorrhœa Alveolaris.*—Use the following:

℞.—Acidi sulphurici, . . . . . ʒ i;

Aquæ destillata, . . . . . ℥ ii;

Sodæ biborat., . . . . . . gr. iv.; or the officinal aromatic sulphuric acid may be used instead. The mode of application is essential to success in the treatment. The best method to apply the remedy is with two slender excavators and a pledget of cotton, (syringing has proven unsatisfactory.) After saturating the pledget, force it down between the tooth and its socket, pumping up and down until the parts are satisfied. Then bathe the gums and teeth with an alkaline wash to neutralize the acid. The next day paint with tincture of iodine. If pus continues to be discharged after ten or twelve days, repeat the operation. It is well to protect the sublingual ducts with a wad of bibulous paper when treating the lower incisors, as an excess of the fluid flowing around underneath the tongue would be liable to produce an eschar. Thorough cleansing of the parts implicated from all extraneous substances is a prerequsite to success. With the treatment thus indicated, a radical cure can be relied upon in most cases. Similar treatment cures gingivitis infallibly. If the mixture is not sufficiently strong, its strength may be increased by the addition of more sulphuric acid.—*Dental Cosmopolitan.*

*Treatment of Gonorrhœa.*—Dr. Haberkorn, in the *Berl. Klin. Wochenschrift*, writes that injections of permanganate of potassa, carbolic acid, sulphate of zinc, and other remedies have all proved more or less insufficient in the treatment of gonorrhœa. After repeated experiments he has found the sulphate of quinine to be a far superior remedy, being prompt in its action and nearly painless. He directs a teaspoonful of the following mixture to be injected three times a day, and retained for some time in the urethra:

| | |
|---|---|
| ℞.—Quiniæ sulphat., . . . . | gr. xv. |
| Acid sulph. dil., . . . . . | ℈j. |
| Glycerinæ, . . . . . . | fl. ʒ vj. |
| Aqua, . . . . . . . | fl. ℥ ij. |

After three days a great improvement took place in all his cases.

*On the Use of Oxide of Zinc in the Treatment of Diarrhœa.*—From the results obtained by M. Gubler, Dr. Henry Puygauthier (*Theses de Paris*, 1874), considers the oxide of zinc as the most prompt and effectual agents in cases of diarrhœa. Four grammes (about one drachm) of the oxide are given during the day, in doses of one gramme every two hours, in wafer-paper. In order to prevent the formation of salts of zinc, and the nausea and vomiting which would ensue, M. Gubler mixes four and a half grammes with fifty centigrammes of bicarbonate of soda, and has obtained with this mixture the best results. Administered in this way, the oxide of zinc loses its nauseating and emetic properties, whilst it retains all its power as an antidiarrhœic.—*London Med. Record,* Dec. 16, 1874.

*Varicose Veins.*—John Marshall (University College Hospital, London,) removed nine inches of a badly varicose internal saphenous vein below the knee. He marked the course of the tortuous vein with ink. Esmarch's elastie bandage was then applied, and an incision was made through the skin from the upper to the lower end of the ink mark. The vein thus exposed was ligated at both ends and entirely removed with forceps and scissors. The operation was performed under the carbolized spray, and Lister's antiseptic method of dressing strictly employed. Patient was discharged from the hospital seven weeks after the operation.—*Braithwaite's Retrospect, July,* 1875.

*Another "Miracle."*—In connection with the famous Belgian case of Louise Leteau, fully described some months ago in the *Reporter*, it may be of interest to notice a case of catalepsy, now under observation at the Hôpital Cochin, Paris. It is that of a hysterical woman, with alternate attacks of paralysis and contraction. The limbs, when put in a certain position, remain in it for a protracted period of time. The woman passes several days in a lethargic state, during which she takes no food, and presents marked insensibility of the skin and mucous membrane. The patient improved under the influence of tonics and food. Had she been in superstitious hands, we should have had another "living miracle."—*Medical and Surgical Reporter.*

*Obstinate Vomiting in Pregnancy Treated by Dilatation of the Os Uteri.*—Dr. E. Copeman (*British Medical Journal*, May 15, 1875,) reports three cases of vomiting during pregnancy, which, after all other means had failed, were completely relieved by dilating the os uteri with the forefinger. In the first place, he was endeavoring to bring on a miscarriage to prevent the death of the woman from exhaustion. Having failed, with the means at his disposal, to do more than dilate the os, he desisted. While waiting for a better instrument, the vomiting ceased and never recurred—the woman going on to the full period of pregnancy—was then delivered of a healthy child, and made a good recovery—*Baltimore Physician and Surgeon.*

*Treatment of Erythema and Intertrigo by Means of Collodion.*—Collodion is a very valuable agent in the treatment of erythema and intertrigo occurring around the genitals and buttocks, and also between the folds of the neck. The mode of its application is to first thoroughly dry the parts, and then quickly, by means of a brush, cover the whole of the irritated and inflamed skin with a layer or collodion. Considerable pain is experienced for a few moments, and it is necessary to hold the patient till the ether has evaporated. In intertrigo of the genitals, the ordinary diaper must be dispensed with, and instead a folded cloth or diaper placed antero-posteriorly, so as to retain the urine and fæces.—*New York Medical Journal.*

*Homeopathic Medicines Must Not Be Shaken.*—We find the following paragraph in an exchange: "Homeopathic physicians are cautioned, in the *Druggist's Journal*, not to spring from their carriages so as to alight forcibly on the ground, as the jar to the medicine in their pockets increases its power so much as to make it dangerous to patients. A Boston man was nearly killed from this cause not long ago."

We suspect this was published for a joke. But it is not the only thing in homeopathy which would be taken for a joke anywhere else. If the reader will refer to Hahnemann's *Organon*, he will find the statement plainly made, that for the reason alleged, homeopathists should not carry their medicines in their pockets, as he had himself observed that by the agitation thus produced, they had acquired a dangerous potency.—*Pacific Medical and Surgical Journal.*

*Rheumatism Treated by a Non-Nitrogenous Diet.*—Dr. Andrews (*Doctor*, May, 1875,) says that he placed eight cases of acute rheumatism upon a non-nitrogenous diet with the effect of obtaining better results than from any other plan of treatment. On the average the pains disappeared in 3.75 days—the longest time being five, the shortest two days. Dr. Moss, of the Royal Naval Hospital, Vancouver's Island, applied the treatment to chronic rheumatism. His success was not great. The diet consisted of arrowroot cakes made with butter, and sometimes with sugar or treacle. The bowels became regular, the rheumatic pains lessened, and the dyspepsia disappeared.—*Baltimore Physician and Surgeon.*

*Effects of the Inhalation of Chloroform, during Parturition, on the Fœtus.*—It is stated in foreign journals, that Dr. Sweifel, of Strasburg, submitted the placenta to analysis, by distillation, in a case where chloroform had been inhaled, and obtained chloroform, recognized by several accurate tests. He has also found that the urine of new-born children whose mothers had used chloroform reduces Fenling's solution, and has, moreover, observed that the expired air of such children has for several hours a well-marked odor of chloroform. Dr. Sweifel, arguing from these facts, concludes that the chloroform inhaled by the mother passes into the circulation of the fœtus.—*Medical and Surgical Reporter.*

*Sciatica Cured by Eating.*—Dr. J. Pretz, (*Wiener Med. Pressé—Medical Times,*) from his personal experience, states the following: During an attack of sciatica, he for six months tried all the ordinary remedies without avail. Observing that his attacks were slighter after eating, he determined to eat as often as the pain recurred. This was frequently twelve times in twenty-four hours. He constantly improved under this treatment, and in about two months entirely recovered. He afterwards treated two other cases with similar results.—*Baltimore Physician and Surgeon.*

*Treatment of Whooping-Cough* (*American Practitioner.*)—Wild claims that he can cure every case of whooping-cough within eight days by the following treatment: the patient is not to leave his room, and at every access of coughing is to place before his mouth a small piece of cloth folded several times and wet with a teaspoonful of the following solution: ether, sixty parts; chloroform, thirty parts; turpentine, one part.

*Ethiops Mineral.*—Professor Cadet states that the vapors of the sulphuret of mercury have been efficient in protecting from the paludal fever of the Campagna, etc. Equal parts of mercury and flowers of sulphur are boiled in water, in a room, in such quantities that the vapor is not unpleasant.

*Liniment for Scabies.*—Dr. Clemens gives the following formula: take of arsenious acid one grain, carbonate of potash fifteen grains, spirit of soap three drachms, spring water three ounces; the liniment to be rubbed twice daily on the part affected. It does not harm the youngest child.—*Practitioner.*

A SENTENCE of death has been pronounced against a man named Graves, who seduced a young lady in Brockville, Canada, and a Dr. Eric B. Sparham, who, at the desire of the former, produced an abortion, causing her death.

*School for Nurses.*—The Commissioners of Public Charities and Correction, New York, opened a school for Nurses at Charity Hospital, New York, on August 1st.—*N. Y. Medical Journal.*

